# Prediction of Thermostability from Amino Acid Attributes by Combination of Clustering with Attribute Weighting: A New Vista in Engineering Enzymes

**DOI:** 10.1371/journal.pone.0023146

**Published:** 2011-08-10

**Authors:** Mansour Ebrahimi, Amir Lakizadeh, Parisa Agha-Golzadeh, Esmaeil Ebrahimie, Mahdi Ebrahimi

**Affiliations:** 1 Department of Biology & Bioinformatics Research Group, University of Qom, Qom, Iran; 2 Department of Computer Sciences & Bioinformatics Research Group, University of Qom, Qom, Iran; 3 Department of Crop Production & Plant Breeding, College of Agriculture, Shiraz University, Shiraz, Iran; 4 Max-Planck-Institute for Informatics, Saarbrucken, Germany; University of Vermont, United States of America

## Abstract

The engineering of thermostable enzymes is receiving increased attention. The paper, detergent, and biofuel industries, in particular, seek to use environmentally friendly enzymes instead of toxic chlorine chemicals. Enzymes typically function at temperatures below 60°C and denature if exposed to higher temperatures. In contrast, a small portion of enzymes can withstand higher temperatures as a result of various structural adaptations. Understanding the protein attributes that are involved in this adaptation is the first step toward engineering thermostable enzymes. We employed various supervised and unsupervised machine learning algorithms as well as attribute weighting approaches to find amino acid composition attributes that contribute to enzyme thermostability. Specifically, we compared two groups of enzymes: mesostable and thermostable enzymes. Furthermore, a combination of attribute weighting with supervised and unsupervised clustering algorithms was used for prediction and modelling of protein thermostability from amino acid composition properties. Mining a large number of protein sequences (2090) through a variety of machine learning algorithms, which were based on the analysis of more than 800 amino acid attributes, increased the accuracy of this study. Moreover, these models were successful in predicting thermostability from the primary structure of proteins. The results showed that expectation maximization clustering in combination with uncertainly and correlation attribute weighting algorithms can effectively (100%) classify thermostable and mesostable proteins. Seventy per cent of the weighting methods selected Gln content and frequency of hydrophilic residues as the most important protein attributes. On the dipeptide level, the frequency of Asn-Glu was the key factor in distinguishing mesostable from thermostable enzymes. This study demonstrates the feasibility of predicting thermostability irrespective of sequence similarity and will serve as a basis for engineering thermostable enzymes in the laboratory.

## Introduction

The primary structure of a protein is the most important factor in determining enzyme thermostability. This stability can be improved by adjusting external environmental factors including cations, substrates, co-enzymes, and modulators. Considerable attention has been paid to thermostable enzymes. Many industrial applications have been reported for thermostable enzymes because they are more stable and generally better suited to harsh processing conditions [1], [2]. With some exceptions, enzymes present in thermophiles are more stable than those found in their mesophilic counterparts. Further research will allow additional exploitation of thermophiles for biotechnology applications. The cloning of enzymes from thermophiles into mesophilic hosts is especially promising. However, most currently available thermostable enzymes have been derived from mesophiles.

To successfully engineer new proteins, we must discover the factors responsible for enzyme thermostability and determine what differentiates them from mesophilic proteins. Moreover, an understanding of thermostability is of primary importance in the engineering thermostable enzymes. Various methods have been proposed to predictfor predicting the stability of proteins based on amino acid substitutions. In some studies, mutants have been reported to be more thermostable [3]. It has been documented that intrahelical salt bridges are more prevalent in thermostable enzymes. Additionally, the composition of amino acids might be an important factor in stability. Moreover, hydrophobic and charged amino acids are more prevalent in thermophilic proteins [4]. Due to their importance in industrial applications, there has been considerable recent interest in understanding the thermostability of enzymes. A method to convert input data from protein properties into a predicted value for thermostability would be particularly useful.

The amino acid sequence (primary structure) of a protein is the main indicator of its function. However, it is generally agreed that direct prediction of protein characteristics such as thermostability and halostability from the primary amino acid sequence is not possible. Consequently, methods to predict thermostability have focused on tertiary and quaternary structures (i.e., three-dimensional structure and molecular protein volume) [5]. Further advances have been hindered by the difficulties in manipulating these complex features. There is a gap between computational biology results and laboratory applications; in the laboratory, we are limited to simple substitution of a small number of amino acids in the primary protein structure.

Recently, we analysed the performance of different attribute weighting, screening, clustering, and decision tree algorithms to discriminate halophilic and non-halophilic proteins [6]. The results showed that amino acid composition can be used to efficiently discriminate between different halostable protein groups with up to 98% accuracy. These results are possible when an appropriate machine learning algorithm mines a large number of structural amino acid attributes of the primary protein structure [6]. In another recent study, we used data mining algorithms to build a precise model to discriminate P1B-ATPase heavy metal transporters in different organisms based on their structural protein features [7]. Moreover, reliable models to predict the hyperaccumulating activity of unknown P1B-ATPase pumps were developed [7]. A support vector machines (SVMs) algorithm was used to predict the functional class of lipid binding proteins [8]. In our previous study, we observed that supervised decision tree algorithms can be employed for extracting the protein attributes that contribute to the thermostability of a protein [9]. Data mining (machine learning) models may have the potential to link a protein's amino acid structure with its thermostability.

Hundreds or thousands of variables may be included in data mining cases [10]. When very large numbers of variables are utilised, more time may be needed to apply a neural network or a decision tree to the dataset [11]. Because many attributes determine the different characteristics of a protein molecule [12], [13], the majority of time and process resources should be spent in determining which variables to include in the model. Attribute weighting (or feature selection) models create a more manageable set of attributes for modelling by reducing the size of attributes [14].

To organise data into a more meaningful form, clustering algorithms partition the data into groups or clusters according to various criteria [15]. Clustering, commonly called unsupervised learning, may proceed according to some parametric model or by grouping points according to some similarity or distance measures (as in hierarchical clustering algorithms) [16]. A suitable unsupervised algorithm is capable of discovering structure on its own by exploring similarities or differences between individual data points in a data set [17]. K-Means is one of the simplest unsupervised learning algorithms for solving well-known clustering problems. The procedure follows a simple method to classify a given data set through a certain number of clusters (assume k clusters) fixed a priori. Generally, the objective is to define k centroids, one for each cluster [18]. The K-Medoids method uses representative objects as reference points instead of taking the mean value of the objects in each cluster [19]. The support vector clustering (SVC) algorithm is a recently developed unsupervised learning method, which was inspired by support vector machines. Cluster assignment of each data point is the key step in the SVC algorithm [20]. Expectation-Maximization Clustering (EMC) is an effective, popular technique to estimate mixture model parameters (cluster parameters and their mixture weights). The EMC algorithm iteratively refines initial mixture model parameter estimates to better fit the data, and it then terminates at a locally optimal solution [21].

Artificial neural networks are computing systems that simulate the biological neural systems of a human brain [22]. The basic strategy for developing a neural-based model of a given material behaviour is to train a neural network using the results of a series of experiments on the material. Traditionally, the learning process is used to establish proper interconnection weights, and the network is trained to make proper associations between the inputs and their corresponding outputs. Neural networks can be classified into dynamic and static categories [23]. Dynamic networks can also be divided into the following two categories: those that have only feed-forward connections and those that have feedback, or recurrent, connections [24]. The dynamic network's memory and response at any given time depends not only on the current input, but also on the history of the input sequence. Elman neural networks are examples of recurrent neural networks. Elman networks are multiple layer back propagation networks, which also have a feedback connection from the output of the hidden layer to its input [25]. The default method to improve generalisation of neural networks is known as early stopping. In this technique, the available data is divided into two subsets. The first subset is the training set, which is used to compute the gradient and update the network weights and biases. The second subset is the validation set. The test set error is not used during training, but it is used to compare different models. It is also useful to plot the test set error during the training process. Cross validation [26] is used to train and test models on all patterns. There must be no overlap between training and testing sets to evaluate neural networks.

The aim of the present investigation was to determine the most important amino acid attributes that contribute to protein thermostability. We examined a variety of attribute weighting algorithms and various supervised and unsupervised clustering models on a large number (800) of amino acid properties. More importantly, we successfully established an accurate expert system to predict the thermostability of any input sequence. This result was obtained by performing unsupervised clustering on attributes found to contribute to thermostability by suitable attribute weighting algorithm. In the present method, there is no need to utilise any sequence similarity searches or protein tertiary/quaternary features.

## Results

### Data cleaning

The initial dataset contained 2090 records (protein sequences) with 852 protein attributes. Of these records, 75% (1573 records) were classified as T (mesostable) class, and the remainder (517 or 25% of records) were classified as F (thermostable) class. Following removal of duplicates, useless attributes, and correlated features (data cleaning) 2057 records and 794 features remained.

### Attribute weighting

Data were normalised before running the models; it was expected that all weights would be between 0 and 1.

#### Weighting by PCA

The following nine attributes weighed equal to or higher than 0.80: the counts of Asp, Glu, Phe, His, Met, Ser, Val, and Tyr.

#### Weighting by SVM

The following 27 attributes were selected by this model: the frequencies of hydrophilic residues, Gly, Asn, Tyr, Asp – Pro, Glu – Ile, Glu – Gln, Met – Leu, Met –Thr, Asn – Asn, Pro – Tyr, Thr – Lys, Trp – Leu and Tyr – Val, the counts of Gln, Glu – Gln, Lys – Gln, Asn – Asn, Trp – Leu and frequency of other residues, and the percentages of Thr and Val.

#### Weighting by Relief

The following 7 attributes showed weights higher than 0.50 when this model applied to the dataset: the counts of Met – Gln and the frequency of other residues, the percentage of Gln and the frequencies of hydrophilic residues, Asn, Asn – Ile, and Gln – Leu.

#### Weighting by Uncertainty

The following protein attributes resulted in weights higher than 0.50: the frequency of hydrophilic residues, the count of other residues, the count of Gln, the frequency of Asn, the frequency of Arg, the percentage of Glu, the percentage of Gln, the count of Asn – Asn, the count of Gln – Asn, the frequency of Asn – Asn, and the frequency of Gln – Asn.

#### Weighting by Gini index

The count of Met – Tyr was the only attribute with weight equal to 1.00.

#### Weighting by Chi Squared

The following five attributes were weighted higher than 0.50: The frequency of hydrophilic residues, frequency of Asn, the count of other residues, the percentages of Glu, and the percentages Gln.

#### Weighting by Deviation

The following 27 attributes were weighted higher than 0.50: count of ten dipeptides (His – Cys, His –Trp, Met – Cys, Met – Trp, Gln – Cys, Gln –Trp, Trp – Cys, Trp – His, Trp – Met, and Trp – Trp) and the frequencies of seventeen dipeptides (His – Cys, His – Trp, Lys – Cys, Met – Cys, Met – Trp, Gln – Cys, Gln – Trp, Arg – Cys, Trp – Cys, Trp – His, Trp – Met, Trp – Pro, Trp – Gln, Trp – Thr, Trp – Trp, Trp – Tyr, and Tyr – Cys).

#### Weighting by Rule

The following attributes were weighted equal to or greater than 0.50 when rule algorithm run on dataset: aliphatic index, non-reduced absorption at 280 nm, the percentage of Thr, the frequency of Asn – Asn and the contents of Asn – Asn, Pro – Gln, and Gln – Asn.

#### Weighting by Correlation

Among the protein attributes that were weighted equal to or greater than 0.50, the following 11 were selected: the frequency of hydrophilic, the count of other residues, the count of Gln, the frequency of Asn, the frequency of Arg, the percentage of Glu and Gln, the count of Asn – Asn, the count of Gln – Asn, the frequency of Asn – Asn, and the frequency of Gln – Asn.

#### Weighting by Information Gain

When this algorithm was applied to the dataset, only the following 3 features had weights equal to or higher than 0.50: the frequency of Glu, the percentage of Asn, and the percentage of Val.

Table 1 highlights the most important attributes that were confirmed, by different weighting algorithms, to be involved in thermostability.

**Table 1:** The most important protein attributes (features) selected by different attribute weighting algorithms.

Attribute	The number of attribute weightings that indicate the attribute is important
The percentage of Gln	7
The frequency of hydrophilic residues	7
The count of other residues	7
The percentage of Glu	6
The frequency of Asn	6
The frequency of Asn- Gln	4
The count of Asn-Asn	4
The frequency of Asn-Asn	4
The count of Gln	3
The Percentage of Thr	3
The percentage of Val	2
The frequency of Arg	2
The count of Pro-Gln	2
The count of Lys-Gln	2

This table presents the number of algorithms that selected the attribute. Weighting algorithms were PCA, SVM, Relief, Uncertainty, Gini index, Chi Squared, Deviation, Rule, Correlation, and Information Gain.

### Unsupervised clustering algorithms

Four different unsupervised clustering algorithms (K-Means, K-Medoids, SVC and EMC) were applied on ten datasets created using attribute selection (weighting) algorithms. Some models, such as the application of the EMC algorithm on datasets created by Chi squared, Gini Index, Information Gain, Relief, Rule or PCA were unable to differentiate T (mesostable) proteins from F (thermostable) proteins (all proteins were selected as F class) (Table 2). Application of the EMC algorithm to the Deviation dataset was unable to assign any protein into its correct class. Some other algorithms, such as the application of K-Medoids on the Chi Squared dataset, assigned most of the T class proteins to the F class. Interestingly, just application of EMC clustering method to the uncertainly and correlation datasets was able to categorise proteins into the correct clusters with 100% accuracy. On the other words, combination of EMC clustering method with either uncertainly or correlation attribute weighting selected the right classes of T for 1544 mesostable proteins and F for 513 thermostable proteins (Table 2).

**Table 2:** Clustering of 10 datasets (generated after performing 10 attribute weighting algorithms) into T (mesophile) and F (thermophile) classes by four different unsupervised clustering algorithms (K-Means, K-Medoids, SVC and EMC).

	Chi Squared		Correlation		Deviation		Gini Index		Information Gain		Relief		Rule		PCA		SVM		Uncertainty	
	T	F	T	F	T	F	T	F	T	F	T	F	T	F	T	F	T	F	T	F
**K-Means**	1461	596	1810	247	1222	835	1452	605	1333	724	1603	454	1601	456	434	1623	1076	981	372	1685
**K-Medoids**	487	1570	1521	536	104	1953	1570	487	1152	905	583	1474	1652	405	1768	289	892	1165	939	1118
**SVC**	363	1688	1701	328	1705	6	363	1688	570	1487	529	1324	1561	4	631	1426	0	2057	1089	947
**EMC**	0	2057	1544	513	0	0	0	2057	0	2057	0	2057	4	2053	0	2057	0	2057	1544	513

The actual numbers of T (mesostable) and F (thermostable) classes in the original datasets were 1544 and 513, respectively. The highest accuracy (100%) was observed when the EMC clustering method was applied to datasets generated by Correlation and Uncertainty attribute weighting algorithms that highlighted in the table.

### Supervised Clustering

#### Decision Tree

Of the 37 different decision tree models applied on the datasets, just 8 of them (run on FCdb) resulted in trees with roots and leaves; the others models were not able to produce such trees. The following models produced trees with roots and leaves: two Decision tree models (with Gain Ratio and Information Gain criteria), two Random Forest models (with Gini Index and Accuracy criteria), two Decision Tree Parallel models (on Gain Ratio and Information Gain criteria), one ID3 Numerical Parallel (on Gain Ratio) and one Decision Stump model (on Gain Ratio criterion).

The simplest tree was produced by the Decision Stump model on the Gain Ratio criterion; the frequency of Gln – Asn was the sole protein attribute used to build this tree. If the frequency was greater than 0.500, the protein belonged to the F (thermostable class; otherwise, the protein belonged to the T (mesostable) class. The Parallel Decision Tree model induced a two-level tree; the percentage of Glu was the most important feature to build the tree. Specifically, when the value for this feature was higher than 0.322, the proteins fell into the T class, but when the value was equal to or lower than 0.322 and the frequency of hydrophilic residues was higher than 0.550, the enzymes belonged to the F class; otherwise the protein was assigned to the T class.

**Figure 1:**
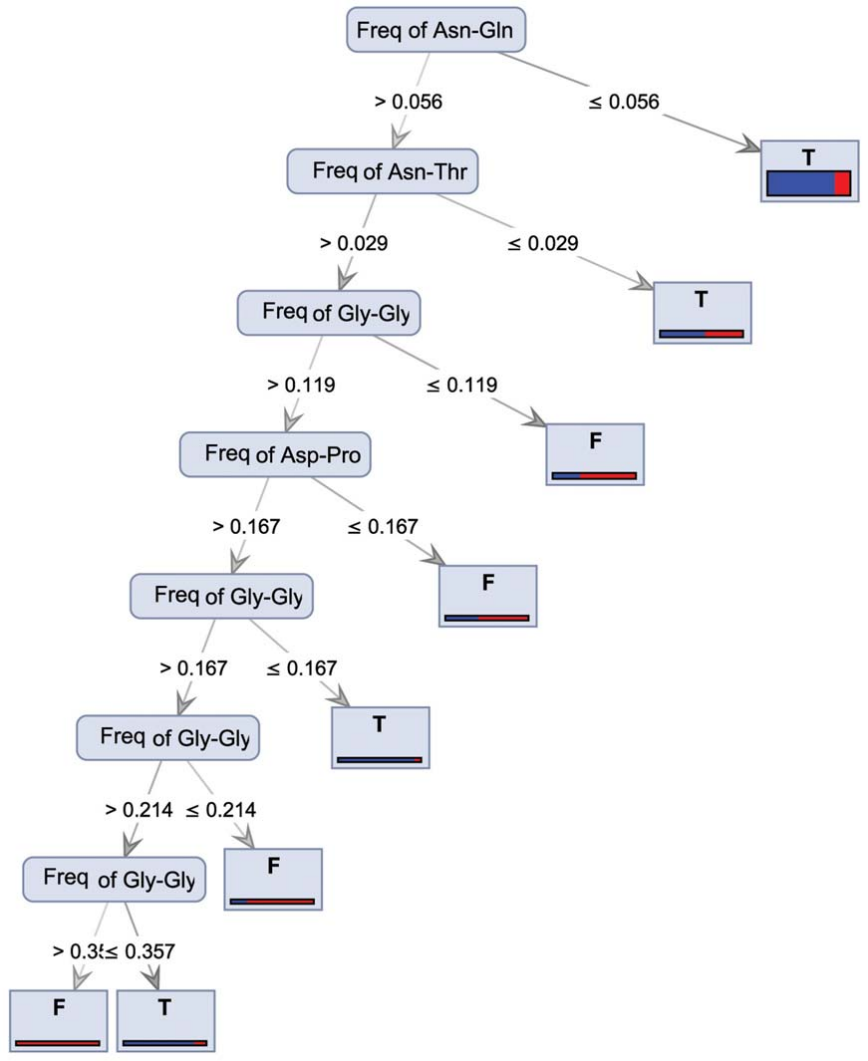
Random Forest decision model on Gini Index criterion. The frequency of Asn – Gln was the most important attribute used to build the tree. The frequencies of Asn – Thr, Gly – Gly, and Asp – Pro were the other features used to build the rest of tree. T: mesostable and F: thermostable.

The frequency of Asn – Gln was the most important attribute used to build the tree when the Random Forest model on Gini Index criterion was applied to the dataset (Figure 1). When the value for this feature was less than or equal to 0.050, the enzymes fell into the T class; if the value was higher than 0.050 and the frequency of Asn – Thr was less than or equal to 0.029, the proteins again belonged to the T class. The frequency of Gly – Gly and Asp – Pro were the other features used to build the rest of tree. This tree was the best model to demonstrate the importance of dipeptides in thermostability.

In the tree produced by the Random Forest (on Accuracy criterion), the following protein attributes were used to build the tree: the count of Met – Cys, the count of Asn – Gln, the weight and the frequency of oxygen. Decision Trees on Information Gain and Gain Ratio criteria, ID3 Numerical Parallel (on Gain Ratio) and Parallel Decision Tree (on Information Gain criterion) induced more complicated trees.

**Figure 2:**
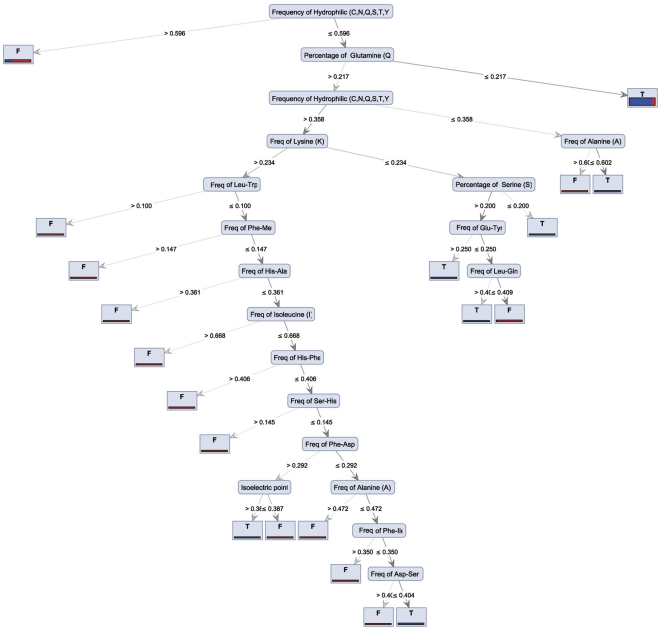
Decision Tree on Gain Ratio model. As can be inferred from the figure, modulation of Glutamine (Gln) content and frequency of hydrophilic residues are the most important protein attributes to distinguish mesostable (T) from thermostable proteins (T).

Figure 2 presents the Decision Tree on Gain Ratio model. As may be inferred from the figure, Glutamine (Gln) content and frequency of hydrophilic residues were the most important protein attributes in distinguishing mesostable (T class) from thermostable (F class) proteins. Higher frequencies of hydrophilic residues (>0.596) generate thermostable proteins, while lower frequencies of hydrophilic residues (<0.596) in combination with low Gln (<0.217) generate mesostable proteins.

#### Neural Networks

In feed-forward model, the best overall accuracy (90% ) was obtained when the number of hidden layers was 3 and the number of neurons in layers was 50, 20, and 10; the accuracy of true and false sections were 85% and 93%, respectively (Table 3). With 4 hidden layers (50, 25, 10, and 5 neurons in hidden layers, respectively), the best overall true and false accuracies in Elman neural networks were 91%, 0.83% and 0.95%, respectively (Table 3). Of the 794 features, 27 were ranked as important contributors to protein thermostability. In networks with 2 hidden layers and 40 or 20 neurons in hidden layers (Table 3), the feed-forward network ran on selected features; the best overall true and false accuracies were 91%, 83% and 95%, respectively. The best results in Elman neural networks on selected features (27 features) were 91%, 95% and 85% for overall true and false accuracies, respectively, for 2 hidden layers and 10 and 5 neurons (Table 3). A detailed result of a 10-fold cross validation of this neural network is presented in Table 4.

**Table 3:** Topologies and overall, true, and false accuracies of the best neural networks run on whole database (with 794 features) and step-wised feature selected database (with 27 features).

Type of neural network	Number of Hidden Layers	Number of Neurons in layer				Per cent of accuracy in predicting thermostable proteins	Per cent of accuracy in predicting mesostable proteins	Per cent of Overall accuracy
		1	2	3	4			
Feed-forward (27 features)	2	40	20			0.84	0.95	0.91
Elman (27 features)	2	10	5			0.84	0.95	0.91
Feed-forward (794 features)	3	50	20	10		0.85	0.93	0.90
Elman (794 features)	4	50	25	10	5	0.83	0.95	0.91

**Table 4:** Ten-fold cross validation of Elman neural network (2 hidden layers with 10 and 5 neurons in each layer) run on a dataset with selected features (with 27 features) presenting overall, mesostable, and thermostable prediction accuracies.

Run	Size of training set	Size of test set	Per cent of accuracy in predicting thermostable proteins	Per cent of accuracy in predicting mesostable proteins	Per cent of Overall accuracy
1	1851	206	0.81	0.85	0.83
2	1851	206	0.76	0.92	0.86
3	1851	206	0.89	0.91	0.90
4	1851	206	0.89	0.96	0.93
5	1851	206	0.90	1.00	0.96
6	1851	206	0.74	0.99	0.89
7	1851	206	0.89	0.96	0.93
8	1851	206	0.84	0.97	0.92
9	1851	206	0.84	0.98	0.92
10	1854	203	0.88	0.93	0.91
**Average**			**0.84**	**0.95**	**0.91**

#### Testing the performance of the best neural networks in predicting the thermostability of new sequence

To evaluate the efficiency of feed-forward and Elman neural networks in predicting the thermostability of other proteins, a dataset of 65 proteins with known thermostability (approximately half of the proteins had temperatures greater than 80°C, and the other half had temperatures less than 80°C) was prepared and the same features (794) were calculated. Then the best neural networks from each category (feed-forward with 2 hidden layers and 40 or 20 neurons in hidden layers and Elman with 2 hidden layers and 10 and 5 neurons in hidden layers) were run on this dataset. The results showed that the accuracies of both neural networks in detecting the correct temperatures for the proteins with optimum temperature higher than 80°C (thermostable group) were approximately 80%. Their accuracies in detecting the optimum temperature lower than 80°C (mesostable group) were higher than 90%. No significant (p>0.95) difference was seen between neural networks running with and without a feature selection algorithm (Table 5).

**Table 5:** Accuracies of the best neural networks gained found in this work in predicting the right temperature for a dataset of 65 new proteins with known temperature.

Dataset without feature selection (all 794) features		
Accuracy%	Feed-forward with 3 hidden layer	Elman with 4 hidden layer
Thermostable proteins	78.46	80.00
Thermostable proteins	93.48	97.83
Overall	85.97	88.91
**Dataset with feature selection (just selected 27) features**		
Thermo-stable proteins	78.46	78.46
False	95.65	95.65
Overall	87.05	87.05

## Discussion

Thermostable enzymes are best suited to harsh conditions, and, thus, there is significant interest in engineering these enzymes for industrial and biotechnical applications [27]. The activity levels of thermostable enzymes are known to increase with increasing temperature. However, at a sufficiently high temperature, inactivation starts to occur [28]. A method to discriminate between thermophilic and mesophilic proteins would be extremely helpful in designing stable proteins [29]. Different models have been proposed to determine the most important attributes that contribute to the stability of proteins at higher temperatures. The models have utilised the crystal structure of thermostable proteins [30], logistic model tree extraction [31], mutant position [3], machine learning algorithms [32], characteristic patterns of codon usage, amino acid composition and nucleotide content [33], disulphide bridge [27], analyses of three-dimensional structures [34], salt bridges [35], aromatic interactions [36], content of Arg, Pro, His, Try [37], the isoelectric points [38], hydrophobicity [39] and the content of electric charges [4]. In the present investigation, we aimed to determine the most important feature contributing to the stability of proteins in harsh thermal conditions. Various modelling techniques were applied to study more than 800 attributes of mesophilic and thermophilic proteins.

When the number of variables or attributes is sufficiently large, the ability to process units is significantly reduced. Data cleansing algorithms were used to remove correlated, useless or duplicated attributes which results in a smaller database [40], [41]. About 10% of the attributes discarded when these algorithms were applied on the original dataset.

Each attribute weighting system uses a specific pattern to define the most important features. Thus, the results may be different [42], as has been highlighted in previous studies [9], [43]. The frequency of hydrophilic residues, the percentage of Gln, and the count of other residues were the most important features to distinguish between mesostable and thermostable enzymes, as defined by 70% of the attribute weighting algorithms. This finding agrees with previous reports. Furthermore, these results confirm the importance of hydrophilicity in allowing tight folding of the proteins and increasing their capacity to resist high temperatures [44]. It has also been demonstrated that the thermostable dehydrins in plant mitochondria are highly hydrophilic, and their accumulation in some species, such as wheat and rye, induce increased thermostability. It has been hypothesised that hydrophilicity stabilises proteins in the membrane or in the matrix when temperature increases [45]. Site-directed mutagenesis has been used to understand thermostability of xylanase enzymes, which confirms the importance of hydrophilicity and salt- bridge [35]. The importance of hydrophobicity (not hydrophilicity) has been highlighted in some studies [32], [39]. However, in a prominent study in this field, the hydration entropy was shown to be a major contributor to the stability of surface mutations in helical segments; these results confirmed that the inverse hydrophobic effect was generally applicable only to coil mutations [3]. An unusually large proportion of surface ion pairs involved in networks that cross-link sequentially separate structures on protein surfaces and an unusually large number of solvent molecules buried in hydrophilic cavities between sequentially separate structures in the protein core of thermostable proteins have been reported [30]. An increase in the number of polar amino acids (such as the count of Gln, as confirmed in this study) may contribute to an increase in the hydrophilic properties of thermostable proteins.

Recent studies have shown that amino acid composition, especially the presence of some polar amino acids such as Gln, exerts a distinguishable effect on thermostability [33]. The most remarkable effect was a two-fold decrease in the frequency of Gln residues among thermophiles [46]. In another study [47], it was shown that the frequency of Gln plays an important role in enzyme thermostability, and our results confirmed this finding [48]. A large number of polar molecules, which may increase molecular hydrophilicity, have been found in the crystal structure of thermostable beta-glycosidase [30]. It has been proposed that amino acid substitution (toward polar amino acids such as Gln) changes protein stability in harsh thermal conditions and might be useful for protein engineering of novel proteins with increased stability and altered function [3]. It has also been noted that thermophiles possess more charged residues, such as Glu, and more hydrophobic residues, as compared to mesophiles [32]. A direct correlation between higher optimum pH and an increase in the number of electrically charged residues, such as Arg, has been observed [37]. This may explain why the number of Gln had higher weights in the applied attribute weighting algorithms we utilised in this study. The roles of other protein residues in enzymes thermostability have been reported previously [49], [50], [51]. Therefore, thermostable proteins and enzymes can be distinguished from mesostable proteins by their amino acid composition, which results in functional adaptation [33]. Previous studies have shown that thermostable enzymes possess significantly higher numbers of certain amino acids, including up to twice as many Gln residues [33]. Additionally, there are significant differences (p<0.01) in the number of certain amino acids (including Gln) present in mesostable and thermostable enzymes [32].

So far, there has been little discussion about the role of dipeptides in protein function. Our recent study has already demonstrated that specific dipeptides play the central role in protein halostability [6]. Furthermore, the role of dipeptides, such as Asp-Gln, in thermostability has been shown in this study. In the Random Forest Decision Tree, the frequency of Asn – Gln was the most important attribute to discriminate between halotolerant and halo-sensitive proteins (Figure 1).

Unsupervised clustering algorithms have been widely employed in a variety of areas in the biological sciences, including diagnostics and image processing [52], EST [17], cancer detection [53], promoter analysis [17], gene and protein bioinformatics [29], [35]. Here, we used four different unsupervised clustering methods (K-Means, K-Medoids, SVC and MEMC) on 10 datasets created from protein attributes, which were assigned high weights. The performances of these algorithms varied significantly (Table 2). Some were unable to assign even a single T protein into the correct class (for example, the EMC algorithm, when applied to most datasets, except Correlation and Uncertainty). Some methods could not put F proteins into the correct class (such as SVC algorithm on Deviation dataset). The results showed that the EMC algorithm was able to classify T and F proteins into the correct classes when runs on the Correlation and Uncertainty datasets. When EMC algorithm combined with Correlation and Uncertainty datasets, the numbers of proteins in each class were exactly the same as in the original dataset, which represents 100% accuracy of this algorithm on the mentioned datasets. To our knowledge, this is the first report of the application of these algorithms to classify thermostable and mesostable proteins. Unsupervised clustering methods are preferred for prediction because they are capable of discovering structure on their own without the need to target variable by exploring similarities and differences between individual data points in a given data set.

This study exploited 10 different attribute weighting methods (including PCA, SVM, Relief, Uncertainty, Gini index, Chi Squared, Deviation, Rule, Correlation, and Information Gain) before importing the data to the above mentioned clustering algorithms. Performance of a clustering algorithm such as EMC can vary from 0.0% to 100%, depending upon which attribute weighting algorithm had summarised the attributes (features) of the dataset prior to running the clustering algorithm (Table 2). This finding highlights the importance of testing different attribute weighting algorithms in biological studies; particular attribute weighting may be unique to each biological case.

Both neural networks were suitable for determining the most important features that contribute to protein thermostability, and no significant differences (p>0.95) were found between neural networks applied here. As mentioned earlier, feed-forward networks are simpler and impose smaller burdens on processors. In this study, we found that the time required for a computer server to run a feed-forward network was at least one third the time for Elman networks. Prior application of a stepwise regression feature selection algorithm showed that the number of variables can be reduced without causing any significant difference in neural networks' accuracies. The use of stepwise regression is highly recommended to minimise the processing time.

Our results show no significant difference (p>0.95%) in overall accuracies of the neural networks (feed-forward and recurrent) tested here. The neural networks resulted in accuracy values slightly higher than 90%, and feature selection modelling did not increase overall accuracies. In contrast, the accuracies of the Elman neural networks applied to the feature selected dataset increased 10% (from 85% to 95%), and its false accuracies decreased to the same extent, demonstrating better efficiency in detecting the correct temperatures.

The best neural networks (feed – forward with 2 hidden layers and 40 and 20 neurons in each hidden layer and Elman with 2 hidden layers and 10 and 5 neurons in each hidden layer) were tested on a separate new database of 65 proteins with known optimum temperatures. The same patterns were observed with total accuracies around 80% and better performances in detecting optimum temperatures lower than 80°C. This result may be due to the ratio of thermostable to mesostable false proteins. The models in this study may be employed to predict the optimum temperature of any new protein sequence.

The major achievement of this study was the prediction of the thermostability of any input protein sequence based on its amino acid composition. These predictions did not require similarity searches or gathering information about the complex, expensive, and time-consuming features of the tertiary and quaternary protein structure. The developed models can be further embedded in web-based data banks to predict protein thermostability from protein sequences. As a result, one can obtain an accurate estimate of increased thermostability from a protein sequence before beginning laboratory activity. In addition, these models provide a new avenue for engineering thermostable enzymes based on the important attributes in this study.

The current findings add to the growing body of literature on engineering thermostable enzymes, which are urgently needed by many industries. The methods used in this study may be applied to broader research areas such as cancer research, prediction protein function, and subcellular localisation. For example, we showed that the above methods can be efficiently employed to distinguish between different protein attributes found in malignant and benign breast cancer proteins and to distinguish the proteins found in different stages [54].

## Materials and Methods

Protein sequences (2090) were extracted from the UniProt Knowledgebase (Swiss-Prot and Tremble) database. More than 63% of the extracted proteins were enzymes. They were categorised into two groups: 1573 or 75% to T (optimum temperature<70°C, mesostable enzymes) and 517 or 25% to F (optimum temperature>70°C, thermostable enzymes). Eight-hundred and fifty-two protein attributes or features such as length, weight, isoelectric point, count and frequency of each element (carbon, nitrogen, sulphur, oxygen, and hydrogen), count and frequency of each amino acid, count and frequency of negatively charged, positively charged, hydrophilic and hydrophobic residues, count and frequency of dipeptides, number of α-helix and β-strand, and other secondary protein features were extracted using various bioinformatics tools and softwares from ExPASy site http://www.expasy.org and CLC bio software (CLC bio, Finlandsgade 10–12, Katrinebjerg 8200 Aarhus N Denmark).

All features were classified as continuous variables, except optimum temperature and N-terminal amino acids, which were classified as categorical. A dataset of these protein features was imported into Rapid Miner (RapidMiner 5.0.001, Rapid-I GmbH, Stochumer Str. 475, 44227 Dortmund, Germany), and the optimum temperature (categorised as T and F) was set as the target or label attribute.

Then, the steps detailed below were applied to the dataset.

### Data cleaning

Duplicate features were removed by comparing all examples with each other on the basis of the specified selection of attributes (two examples were assumed equal if all values of all selected attributes were equal). Next, useless attributes were removed from the dataset. Numerical attributes which possessed standard deviations less than or equal to a given deviation threshold (0.1) were assumed as to be useless and removed. Finally, correlated features (with Pearson correlation greater than 0.9) were omitted. After cleaning, the number of attributes and records decreased to 794 and 2057 respectively; this database was labelled the final cleaned database (FCdb).

### Attribute Weighting

To identify the most important features and to find the possible patterns in features that contribute to thermostability, 10 different algorithms of attribute weightings were applied to the cleaned dataset (FCdb) as described below.

#### Weight by Information gain

This operator calculated the relevance of a feature by computing the information gain in class distribution.

#### Weight by Information Gain ratio

This operator calculated the relevance of a feature by computing the information gain ratio for the class distribution.

#### Weight by Rule

This operator calculated the relevance of a feature by computing the error rate of a OneR Model on the example set without this feature.

#### Weight Deviation

This operator created weights from the standard deviations of all attributes. The values were normalised by the average, the minimum, or the maximum of the attribute.

#### Weight by Chi squared statistic

This operator calculated the relevance of a feature by computing, for each attribute of the input example set, the value of the chi-squared statistic with respect to the class attribute.

#### Weight by Gini index

This operator calculated the relevance of an attribute by computing the Gini index of the class distribution, if the given example set would have been split according to the feature.

#### Weight by Uncertainty

This operator calculated the relevance of an attribute by measuring the symmetrical uncertainty with respect to the class.

#### Weight by Relief

This operator measured the relevance of features by sampling examples and comparing the value of the current feature for the nearest example of the same and of a different class. This version also worked for multiple classes and regression data sets. The resulting weights were normalised into the interval between 0 and 1.

#### Weight by SVM (Support Vector Machine)

This operator used the coefficients of the normal vector of a linear SVM as feature weights.

#### Weight by PCA (Principle Component Analysis)

This operator used the factors of the first of the principal components as feature weights.

### Attribute selection

After attribute weighting models were run on the dataset, each protein attribute (feature) gained a value between 0 and 1, which revealed the importance of that attribute with regards to a target attribute (optimum temperature of enzymes). All variables with weights higher than 0.50 were selected and 10 new datasets (with 2057 records in each dataset) created. These newly formed datasets were named according to their attribute weighting models (Information gain, Information gain ratio, Rule, Deviation, Chi Squared, Gini index, Uncertainty, Relief, SVM and PCA) and were used to join with subsequent models (supervised and unsupervised). Each model of supervised or unsupervised clustering were performed 11 times; the first time it was run on the main dataset (FCdb) and then on the 10 newly formed datasets (the results of attribute weighting).

### Unsupervised clustering algorithms

The clustering algorithms listed below were applied on the 10 newly created datasets (generated as the outcomes of 10 different attribute weighting algorithms (as well as the main dataset (FCdb).

#### K-Means

This operator uses kernels to estimate the distance between objects and clusters. Because of the nature of kernels, it is necessary to sum over all elements of a cluster to calculate one distance.

#### K-Medoids

This operator represents an implementation of k-Medoids. This operator will create a cluster attribute if it is not yet present.

#### Support Vector Clustering (SVC)

This operator represents an implementation of Support Vector algorithm. This operator will create a cluster attribute if not present yet.

#### Expectation Maximization (EM)

This operator represents an implementation of the EM-algorithm.

### Supervised Clustering

#### Decision Trees

Six tree induction models including Decision Tree, Decision Tree Parallel, Decision Stump, Random Tree, ID3 Numerical and Random Forest were run on the main dataset (FCdb). Each tree induction model ran with the following four different criteria: Gain Ratio, Information Gain, Gini Index and Accuracy. In addition, a weight-based parallel decision tree model, which learns a pruned decision tree based on an arbitrary feature relevance test (attribute weighting scheme as inner operator), was run with 13 different weighting criteria (SVM, Gini Index, Uncertainty, PCA, Chi Squared, Rule, Relief, Information Gain, Information Gain Ratio, Deviation, Correlation, Value Average, and Tree Importance).

#### Neural Network

Feed-forward and Elman (as a type of a recurrent) neural networks were run on the two datasets. One dataset had 794, and the next one had 27 protein features chosen after stepwise feature selection algorithm with various hidden layers in each neural network (Table 3). The stepwise regression feature selection algorithm was applied to identify the attributes that had a strong correlation with enzyme thermostability. The algorithm considered one attribute at a time to determine how well each one individually predicted the target variable. The important value for each variable was then calculated as 1−p, where p is the p value of the appropriate test of association between the candidate predictor and the target variable. When the target value was categorical (as in our datasets), p values were calculated based on the F statistic. This approach allows for one-way ANOVA F testing of each predictor. Otherwise, the p value was based on the asymptotic t distribution of a transformation of the Pearson correlation coefficient. Other models, such as likelihood-ratio, Chi-Square (also tests for target-predictor independence), Cramer's V (a measure of association based on Pearson's Chi-Square statistic), and Lambda (a measure of association that reflects the proportional reduction in error when the variable used to predict the target value) were conducted to check for possible effects of calculation on the feature selection criteria. The predictors were then labelled as important, marginal, or unimportant, when values were >0.95, between 0.95 and 0.90, and <0.90, respectively. Finally, the effect of each selected feature was calculated by stepwise regression on the best neural network tested in this paper. Data in each feature was divided into 10 nearly equal classes, and the accuracy was calculated from the number of correct determined records by the network divided by all the records in each class.

The learning algorithm in all networks was back propagation. Cross validation was used to train and test the model on all patterns with no overlap between training and testing sets. To perform cross validation, all the records (2057) were randomly divided into 10 parts; 9 parts consisted of 206 records, and the last one contained 203 records; nine sets were used for training and the 10th one for testing. The process was repeated 10 times and the accuracy for true, false and total accuracy calculated. The final accuracy is the average of the accuracy in all 10 tests.

#### Testing the efficiency of the developed neural networks with new protein sequences

To evaluate the efficiency of the developed networks in predicting the correct temperatures for other proteins, a dataset of 65 proteins with known thermostability (approximately half of the proteins had temperatures greater than 80°C, and the other half had temperatures less than 80°C) was prepared and the same features (794) were calculated. Then, the best developed neural networks were tested on this dataset to predict thermostability. The calculated accuracy indicates the performance of the developed models in predicting the thermostability of new sequences.

## References

[1] Yeoman CJ, Han Y, Dodd D, Schroeder CM, Mackie RI, Allen IL, Sima S, Geoffrey MG (2010). Thermostable Enzymes as Biocatalysts in the Biofuel Industry.. Advances in Applied Microbiology.

[2] Chantasingh D, Pootanakit K, Champreda V, Kanokratana P, Eurwilaichitr L (2006). Cloning, expression, and characterization of a xylanase 10 from Aspergillus terreus (BCC129) in Pichia pastoris.. Protein Expression and Purification.

[3] Gromiha MM, Oobatake M, Kono H, Uedaira H, Sarai A (2002). Importance of mutant position in Ramachandran plot for predicting protein stability of surface mutations.. Biopolymers.

[4] Yang HM, Yao B, Fan YL (2005). Recent advances in structures and relative enzyme properties of xylanase.. Sheng Wu Gong Cheng Xue Bao.

[5] Dalhus B, Saarinen M, Sauer UH, Eklund P, Johansson K (2002). Structural Basis for Thermophilic Protein Stability: Structures of Thermophilic and Mesophilic Malate Dehydrogenases.. Journal of Molecular Biology.

[6] Ebrahimie E, Ebrahimi M, Sarvestani NR (2011). Protein attributes contribute to halo-stability, bioinformatics approach.. Saline Systems.

[7] Ashrafi E, Alemzadeh A, Ebrahimi M, Ebrahimie E, Dadkhodaei N (2011). Amino Acid Features of P1B-ATPase Heavy Metal Transporters Enabling Small Numbers of Organisms to Cope with Heavy Metal Pollution.. Bioinform Biol Insights.

[8] Lin HH, Han LY, Zhang HL, Zheng CJ, Xie B (2006). Prediction of the functional class of lipid binding proteins from sequence-derived properties irrespective of sequence similarity.. J Lipid Res.

[9] Ebrahimi M, Ebrahimie E (2010). Sequence-based prediction of enzyme thermostability through bioinformatics algorithms.. Current Bioinformatics.

[10] Ye X, Fu Z, Wang H, Du W, Wang R (2009). A computerized system for signal detection in spontaneous reporting system of Shanghai China.. Pharmacoepidemiol Drug Saf.

[11] Gromiha MM, Yabuki Y (2008). Functional discrimination of membrane proteins using machine learning techniques.. BMC Bioinformatics.

[12] Ebrahimie E, Ebrahimi M, Rahpayma M (2010). Investigating protein features contribute to salt stability of halolysin proteins.. Journal of Cell and Molecular Research.

[13] Ashrafi E, Alemzadeh A, Ebrahimi M, Ebrahimie E, Dadkhodaei N (2011). Determining specific amino acid features in P1B-ATPase heavy metals transporters which provides a unique ability in small number of organisms to cope with heavy metal pollution.. Bioinformatics and Biology Insights Accepted.

[14] Thai KM, Ecker GF (2009). Similarity-based SIBAR descriptors for classification of chemically diverse hERG blockers.. Mol Divers.

[15] McLachlan GJ, Bean RW, Ng SK (2008). Clustering.. Methods Mol Biol.

[16] Lin Y, Tseng GC, Cheong SY, Bean LJ, Sherman SL (2008). Smarter clustering methods for SNP genotype calling.. Bioinformatics.

[17] Abeel T, Saeys Y, Rouze P, Van de Peer Y (2008). ProSOM: core promoter prediction based on unsupervised clustering of DNA physical profiles.. Bioinformatics.

[18] Billings SA, Wei HL, Balikhin MA (2007). Generalized multiscale radial basis function networks.. Neural Netw.

[19] Sparks ME, Brendel V (2008). MetWAMer: eukaryotic translation initiation site prediction.. BMC Bioinformatics.

[20] De Bruyne V, Al-Mulla F, Pot B (2007). Methods for microarray data analysis.. Methods Mol Biol.

[21] Waydo S, Koch C (2008). Unsupervised learning of individuals and categories from images.. Neural Comput.

[22] Yang ZR (2009). Neural networks.. Methods Mol Biol.

[23] Fisher J, Piterman N (2010). The executable pathway to biological networks.. Brief Funct Genomics.

[24] Valavanis IK, Mougiakakou SG, Grimaldi KA, Nikita KS (2010). A multifactorial analysis of obesity as CVD risk factor: use of neural network based methods in a nutrigenetics context.. BMC Bioinformatics.

[25] Heider D, Appelmann J, Bayro T, Dreckmann W, Held A (2009). A computational approach for the identification of small GTPases based on preprocessed amino acid sequences.. Technol Cancer Res Treat.

[26] Habashy HO, Powe DG, Glaab E, Ball G, Spiteri I (2010). RERG (Ras-like, oestrogen-regulated, growth-inhibitor) expression in breast cancer: a marker of ER-positive luminal-like subtype.. Breast Cancer Res Treat.

[27] Wakarchuk WW, Sung WL, Campbell RL, Cunningham A, Watson DC (1994). Thermostabilization of the Bacillus circulans xylanase by the introduction of disulfide bonds.. Protein engineering.

[28] Paloheimo M, Mantyla A, Kallio J, Puranen T, Suominen P (2007). Increased production of xylanase by expression of a truncated version of the xyn11A gene from Nonomuraea flexuosa in Trichoderma reesei.. Applied Environmental Microbiology.

[29] Adams MW, Kelly RM (1998). Finding and using hyperthermophilic enzymes.. Trends Biotechnol.

[30] Aguilar CF, Sanderson I, Moracci M, Ciaramella M, Nucci R (1997). Crystal structure of the beta-glycosidase from the hyperthermophilic archeon Sulfolobus solfataricus: resilience as a key factor in thermostability.. J Mol Biol.

[31] Dancey D, Bandar ZA, McLean D (2007). Logistic model tree extraction from artificial neural networks.. IEEE Trans Syst Man Cybern B Cybern.

[32] Gromiha MM, Suresh MX (2008). Discrimination of mesophilic and thermophilic proteins using machine learning algorithms.. Proteins.

[33] Singer GA, Hickey DA (2003). Thermophilic prokaryotes have characteristic patterns of codon usage, amino acid composition and nucleotide content.. Gene.

[34] Bogin O, Peretz M, Hacham Y, Korkhin Y, Frolow F (1998). Enhanced thermal stability of Clostridium beijerinckii alcohol dehydrogenase after strategic substitution of amino acid residues with prolines from the homologous thermophilic Thermoanaerobacter brockii alcohol dehydrogenase.. Protein Sci.

[35] Georis J, de Lemos Esteves F, Lamotte-Brasseur J, Bougnet V, Devreese B (2000). An additional aromatic interaction improves the thermostability and thermophilicity of a mesophilic family 11 xylanase: structural basis and molecular study.. Protein Sci.

[36] Asada Y, Endo S, Inoue Y, Mamiya H, Hara A (2009). Biochemical and structural characterization of a short-chain dehydrogenase/reductase of Thermus thermophilus HB8: a hyperthermostable aldose-1-dehydrogenase with broad substrate specificity.. Chem Biol Interact.

[37] Masui A, Fujiwara N, Imanaka T (1994). Stabilization and rational design of serine protease AprM under highly alkaline and high-temperature conditions.. Appl Environ Microbiol.

[38] Berens S, Kaspari H, Klemme JH (1996). Purification and characterization of two different xylanases from the thermophilic actinomycete Microtetraspora flexuosa SIIX.. Antonie Van Leeuwenhoek.

[39] Miyazaki K, Takenouchi M, Kondo H, Noro N, Suzuki M (2006). Thermal stabilization of Bacillus subtilis family-11 xylanase by directed evolution.. Journal of Biological Chemistry.

[40] Rustici G, Kapushesky M, Kolesnikov N, Parkinson H, Sarkans U (2008). Data storage and analysis in ArrayExpress and Expression Profiler.. Curr Protoc Bioinformatics Chapter.

[41] Fu X, Wang L (2003). Data dimensionality reduction with application to simplifying RBF network structure and improving classification performance.. IEEE Trans Syst Man Cybern B Cybern.

[42] Baumgartner C, Lewis GD, Netzer M, Pfeifer B, Gerszten RE (2010). A new data mining approach for profiling and categorizing kinetic patterns of metabolic biomarkers after myocardial injury.. Bioinformatics.

[43] Bijanzadeh E, Emam Y, Ebrahimie E (2010). Determining the most important features contributing to wheat grain yield using supervised feature selection model.. Australian Journal of crop science.

[44] Barthelemy PA, Raab H, Appleton BA, Bond CJ, Wu P (2008). Comprehensive analysis of the factors contributing to the stability and solubility of autonomous human VH domains.. J Biol Chem.

[45] Borovskii GB, Stupnikova IV, Antipina AI, Vladimirova SV, Voinikov VK (2002). Accumulation of dehydrin-like proteins in the mitochondria of cereals in response to cold, freezing, drought and ABA treatment.. BMC Plant Biol.

[46] Akanuma S, Qu C, Yamagishi A, Tanaka N, Oshima T (1997). Effect of polar side chains at position 172 on thermal stability of 3-isopropylmalate dehydrogenase from Thermus thermophilus.. FEBS Lett.

[47] Ebrahimi M, Ebrahimi E, Ebrahimi M (2009). Searching for patterns of thermostability in proteins and defining the main features contributing to enzyme thermostability through screening, clustering, and decision tree algorithms.. EXCLI Journal.

[48] Vitalis A, Lyle N, Pappu RV (2009). Thermodynamics of beta-sheet formation in polyglutamine.. Biophys J.

[49] Bendova-Biedermannova L, Hobza P, Vondrasek J (2008). Identifying stabilizing key residues in proteins using interresidue interaction energy matrix.. Proteins.

[50] Gromiha MM (2001). Important inter-residue contacts for enhancing the thermal stability of thermophilic proteins.. Biophys Chem.

[51] Kirino H, Aoki M, Aoshima M, Hayashi Y, Ohba M (1994). Hydrophobic interaction at the subunit interface contributes to the thermostability of 3-isopropylmalate dehydrogenase from an extreme thermophile, Thermus thermophilus.. Eur J Biochem.

[52] Balasubramanian D, Srinivasan P, Gurupatham R (2007). Automatic classification of focal lesions in ultrasound liver images using principal component analysis and neural networks.. Conf Proc IEEE Eng Med Biol Soc.

[53] de Souto MC, Costa IG, de Araujo DS, Ludermir TB, Schliep A (2008). Clustering cancer gene expression data: a comparative study.. BMC Bioinformatics.

[54] Ebrahimi M, Ebrahimie E, Shamabadi N (2010). Are there any differences between features of proteins expressed in malignant and benign breast cancers?. J Res Med Sci.

